# Augmenting Functional and Sensorial Quality Attributes of Kefir through Fortification with Encapsulated Blackberry Juice

**DOI:** 10.3390/foods12224163

**Published:** 2023-11-17

**Authors:** Vanja Travičić, Olja Šovljanski, Ana Tomić, Milica Perović, Maja Milošević, Nenad Ćetković, Mirjana Antov

**Affiliations:** 1 Faculty of Technology Novi Sad, University of Novi Sad, Bulevar Cara Lazara 1, 21000 Novi Sad, Serbia; oljasovljanski@uns.ac.rs (O.Š.); anav@uns.ac.rs (A.T.); perovicmilica@uns.ac.rs (M.P.); majamilosevicpuhacin@eurofins.se (M.M.); mantov@uns.ac.rs (M.A.); 2 Medical Faculty, University of Novi Sad, Hajduk Veljkova 3, 21000 Novi Sad, Serbia; nenad.cetkovic@mf.uns.ac.rs

**Keywords:** blackberry, lentil protein isolate, encapsulation, freeze-drying, kefir fortification, storage stability

## Abstract

Kefir is a fermented dairy product claimed to confer many health-promoting effects, but its acidic taste is not appealing to some consumers. Therefore, the aim of this study was to enhance the functional and sensorial quality attributes of kefir through fortification with encapsulated blackberry juice (EBJ). The blackberry juice was successfully encapsulated via freeze-drying using lentil protein isolate (LPI) as the carrier. The encapsulated blackberry juice showed good physicochemical, functional, and morphological properties, as well as microbiological safety for use as a food additive. The kefir was fortified with EBJ in concentrations of 1, 2.5, 5, and 7.5% (*w*/*w*), stored for up to 28 days under refrigeration, and periodically evaluated. Parameters such as the viscosity, titrable acidity, and pH indicate that the kefir fortification did not affect its stability during storage. EBJ significantly increased the antioxidant properties of the kefir, depending on the fortification level. Additionally, all the fortified samples provided more anthocyanins than the daily recommended intake. Microbiological profiling demonstrated that good laboratory practice and hygiene were implemented during the experiments. Finally, the panelists showed that higher EBJ concentrations in the kefir resulted in greater overall acceptability, indicating that this encapsulate has the potential to be a substitute synthetic color additive in the dairy industry.

## 1. Introduction

In developing countries, with rapid urbanization, less availability of time, and more concerns about a healthier diet, consumers are relying on adopting new instant food habits. The new lifestyles have driven the food industry to develop new functional foods and beverages with added health benefits. Antioxidants in fruit juices have become increasingly popular because of their potential health benefits and ability to combat oxidative stress and free radicals, as advertised by their manufacturers [1]. Nowadays, juices made from berries and red fruits represent frequent consumer choices due to their high purported nutritive value and high contents of bioactive compounds [2].

The application of bioactive compounds in the creation of functional food has some challenges, as bioactivity depends on preserving the stability and bioavailability of the active ingredients. Color and flavor are also very important quality parameters that are driving forces of consumers’ acceptance of food. In the present study, blackberries were selected as a source of polyphenolic antioxidants, mostly anthocyanins, due to their diverse health benefits, appealing color, and pleasant flavor. During the processing and storage periods, natural polyphenolics can be negatively affected, as they are easily degradable during processing and storage because of their sensitivity to heat, light, oxygen, and pH [3,4]. Nowadays, one of the emerging trends is their incorporation into food products in encapsulated form. For this purpose, the selection of carriers/wall materials for bioactive compounds is of major importance [5].

Proteins are unique carriers that can efficiently encapsulate bioactive compounds and enable their protection against harsh storage, processing, and gastrointestinal tract (GIT) conditions. Among different plant-based proteins that can be used as encapsulation agents, lentil protein is an excellent choice because of its superior emulsifying properties, low cost, good nutritional value, and low allergen risk [6]. Numerous studies report the encapsulation of different bioactive substances with the usage of lentil proteins. Chang et al. [7] developed lentil protein isolate (LPI)-based microcapsules to encapsulate canola oil with an entrapment efficiency of 88%. Native and heated lentil proteins were effective encapsulation agents for the encapsulation of flaxseed oil [8]. Quiroz et al. [9] demonstrated the ability of lentil proteins to encapsulate annatto extract with encapsulation efficiencies of 69%, where the protein structure and amino acid content were the relevant factors to obtain a high encapsulation capability.

Due to the customer desire for food products that promote good health, kefir consumption has continuously grown. Kefir is a dairy product made from kefir grains that contains a specific combination of bacteria and yeast. It is made by mixing milk with a culture known as “kefir grains,” which results in a creamy consistency, sour flavor, and minimal effervescence [10]. In general, the microbial composition of kefir varies depending on the type and composition of the milk, the culture medium, the temperature, and the fermentation period, as well as the storage conditions. The most common bacteria in kefir are *Lactobacillus*, *Lactococcus*, *Streptococcus*, and *Leuconostoc*, while the most common yeasts are *Saccharomyces*, *Kluyveromyces*, and *Candida* [11]. To achieve high-quality kefir, it is essential to maintain a balanced and diverse community of microorganisms, as they work in symbiosis to ferment milk and produce the desired characteristics. Any deviation from this process or contamination by unwanted pathogenic microorganisms can lead to off-flavors, spoilage, or health risks. Therefore, maintaining aseptic conditions during the fermentation process is crucial to prevent contamination by undesirable bacteria, fungi, and/or yeasts. Furthermore, keeping the right balance between the LAB and yeast populations is challenging, as the overgrowth of one group can alter the flavor and texture of the final product. It is important to emphasize that kefir is a perishable product, and maintaining its quality during storage can be challenging. Probiotic microorganisms may lose viability over time. Moreover, monitoring and maintaining the proper pH throughout the fermentation and storage processes are essential to ensure the microbiological quality of the kefir. To ensure good microbiological quality in kefir production and storage, it is important to use proper sanitation practices, control the fermentation conditions, and consider packaging methods that minimize exposure to oxygen and contaminants [12].

Kefir consumption has been linked with an increase in the number of gut health-beneficial bacteria and yeasts; thus, it can prevent digestion problems. In addition, kefir’s anti-bacterial, anti-fungal, and anti-cancer properties have been approved in some research [13]. Despite all these health advantages, kefir’s acidic flavor does not win over many people, especially young people. To increase kefir consumption among youths, kefir is made with a variety of fruit sauces (strawberry, forest fruit, honey–banana, and mixed fruit) and flavors [14]. To provide attractive color, various anthocyanin-rich fruit and vegetable juices (black mulberry [15], pomegranate [16], strawberry [17], and black carrot [18]) are added to kefir at various concentrations.

Studies on fortified kefirs with encapsulated phytochemicals and natural colorants are limited. Therefore, the main purpose of this research was to develop fortified kefir with encapsulated blackberry juice (EBJ). Four different levels of EBJ (1, 2.5, 5, and 7.5% (*w*/*w*) were added to the kefir formulation after fermentation. During the storage period of 28 days at 4 °C, analyses of the quality parameters, such as the color, rheology, titrable acidity, pH value, total polyphenol content, total anthocyanin content, microbiological profiling, and sensorial properties, were performed to confirm that a quality product was obtained. The current study was designed to (i) advance the scientific knowledge of the use of LPI for the encapsulation of phytochemicals such as polyphenols and anthocyanins from blackberry juice, (ii) evaluate the effect of fortification on the kefir physicochemical and sensorial properties, and (iii) establish the level of added ingredients based on consumer acceptance. This approach may be useful to dairy producers in the strategy of widening their product range considering that the interest in and consumption of functional food is increasing.

## 2. Material and Methods

### 2.1. Chemicals

Folin–Ciocalteu reagent, 2,2-diphenyl-1-picrylhydrazyl radical (DPPH^•^), 2,2′-azinobis 3-ethylbenzothiazoline-6-sulphonic acid (ABTS^•+^), Trolox, and trichloroacetic acid were purchased from Sigma Chemical Co. (St. Louis, MO, USA). Methanol, ferric chloride was obtained from J.T. Baker (Deventer, Holland). Methanol and acetic acid were obtained from JT Baker (Deventer, Holland). Manganese (IV) oxide was obtained from Alfa Aesar (Karlsruhe, Germany). Other chemicals and solvents used were of the highest analytical grade. Ultrapure water was produced using an Elix_3 Millipore water purification system coupled to a Milli-Q module (model Advantage10) (Millipore, Molsheim, France).

### 2.2. Plant Material

Berries were washed and the juice was extracted with a laboratory juicer (model JC400E, Gorenje, Velenje, Slovenia). Green lentils (*Lens culinaris*) were purchased locally, ground in an electric mill, and sieved through an 18-mesh sieve. The lentil flour was stored in a sealed plastic bag at 4 °C until use.

### 2.3. Protein Extraction

Protein isolates were obtained from green lentil flour via alkaline extraction followed by isoelectric precipitation and freeze-drying. Lentil flour was mixed with distilled water in a 1:10 (*w*/*v*) mixing ratio, and the pH was adjusted to 9 with 0.1 M NaOH solution. The alkaline extraction lasted 1 h at 25 °C with constant stirring (400 rpm) on a magnetic stirrer (RH basic, IKA, Staufen, Germany). After extraction, solids were removed via centrifugation (9000× *g* for 10 min at 4 °C, Sorvall RC-5B, DuPont Instruments, Boston, MA, USA). The supernatant was subjected to isoelectric precipitation by adjusting the pH to 4.2 using 0.1 M HCl. Centrifugation was repeated under the same conditions in order to collect the precipitated protein. Precipitate was redissolved in distilled water and freeze-dried at −40 °C for 24 h (Alpha 2–4 LSCbasic, Martin Christ, Osterode am Harz, Germany).

### 2.4. Blackberry Juice Encapsulation with Lentil Proteins

The freeze-drying encapsulation process, which is described by Čakaraveć et al. [19], implied the following steps: The carrier LPI was dispersed in blackberry juice in a core–carrier ratio of 1:20 *w*/*v* and homogenized with an Ultra-Turrax homogenizer at 11,000 rpm for 5 min, followed by shaking on a laboratory shaker at 400 rpm for 15 min. The prepared formulation was kept overnight at −20 °C in a freezer, and then freeze-dried (Christ Alpha 2–4 LSC, Martin Christ, Osterode am Harz, Germany) at −45 °C for 48 h to ensure complete drying. The freeze-dried encapsulate was stored at −20 °C pending further use.

### 2.5. Characterization of Lentil Protein Isolate (LPI) and Encapsulated Blackberry Juice (EBJ)

#### 2.5.1. Physicochemical and Color Properties

The water activity, or a_w_ value, was determined via a LabSwift-aw meter (Novasina, Lachen, Switzerland) at 25 °C. The moisture content was determined using the air oven method at 105 °C, until a constant weight was obtained. For hygroscopic moisture analysis, about 2 g of encapsulates were placed at 25 °C in an airtight plastic container filled with NaCl-saturated solution (75.29% RH). The hygroscopic moisture was measured after one week, and the result is expressed as grams of moisture per 100 g of dry solids (g/100 g). Solubilities of the LPI and EBJ were determined in distilled water at pH 9 according to Perović et al. [20]. The water-holding capacity (WHC) and oil-holding capacity (OHC) of the LPI and EBJ were determined according to Tan et al. [21]. The WHC and OHC are presented as grams of water or oil held by 1 g of sample. To define the encapsulate flow properties, the bulk density (Db), tapped density (Dt), compressibility ratio (CR), and Hausner ratio (HR) were determined according to Šeregelj et al. [22]. Further, the classification of the encapsulate flowability and cohesiveness was made as outlined by Jinapong et al. [23]. The measurements of the color properties were taken with a Minolta reflectance colorimeter (Minolta ChromaMeter CR-300, Minolta, Osaka, Japan) considering the CIELab color system (L*, a*, and b*). Chroma (C*) was calculated manually via the equation √(a^2^ + b^2^).

#### 2.5.2. Morphological Properties

The morphological properties of the lentil protein isolate and encapsulate were assessed via a HITACHI TM 3030 scanning electron microscope (Hitachi, Chiyoda, Tokyo) at a custom magnification.

#### 2.5.3. Phytochemical Content and Antioxidant Properties

For the determination of the total polyphenol content (TPC), 100 mg of LPI and EBJ were mixed with 2 mL ethanol, acetic acid, and water (50:8:42) on vortex for one minute, and then centrifuged at 4000 rpm for three minutes. The liquid part was collected and filtered through a 0.45 μm filter. The TPC was determined spectrophotometrically via the Folin–Ciocalteu method adopted to microscale [24]. Results are expressed as milligrams of gallic acid equivalents per 100 g (mg GAE/100 g).

For the determination of the total anthocyanin content (TAC,) the same extraction procedure as for the total polyphenol content was used. The TAC was determined spectrophotometrically according to the pH differential method described by Lee et al. [25]. Results are expressed as milligrams of cyanidin-3-glycoside per 100 g (mg CyGE/100 g).

The encapsulation efficiencies of the polyphenols and anthocyanins (EE TPC and EE TAC, respectively) were determined according to the Tumbas Šaponjac et al. [26] protocol. The quantification of the polyphenols and anthocyanins was performed following the previously mentioned spectrophotometric procedure. The EE TPC (EE TAC) was calculated by using the following equation:

EE TPC = [(CPC − SPC)/CPC] × 100

EE TAC = [(CAC − SAC)/CAC] × 100

where CPC (CAC) is the polyphenols (anthocyanins) inside the core of the encapsulate, while SPC (SAC) is the surface polyphenol (anthocyanin) content. The control sample was the carrier without the blackberry juice.

The analysis of the antioxidant activity, expressed as micromole Trolox equivalent (TE) per 100 g of LPI or EBJ, was performed via three methods adapted to microscale: the 2,2-diphenyl-1-picrylhydrazyl (DPPH) method, the 2,2-azino-bis-3-ethylbenzothiazoline-6 acid (ABTS) method by Tumbas Šaponjac et al. [27], and the reducing power (RP) method by Oyaizu [28].

#### 2.5.4. Microbiological Profiles

The microbiological profiles of the LPI and EBJ were determined via ISO standardized analyses: aerobic mesophilic bacteria (ISO 4833-1:2013) [29], yeast and mold (ISO 21527-1:2008) [30], Enterobacteriaceae (ISO 21528-2:2017) [31], and *Clostridium perfringens* (ISO 7937:2004) [32]. For each analysis, the samples were mixed with Peptone Water solution (HiMedia, Mumbai, India) in a ratio of 1:10 using an EasyMIX blender (BioMerieux, Marcy-l’Étoile, France). Subsequently, a series of dilutions were prepared, and aliquots were streaked into a (non)selective medium following the mentioned standards. After all incubation steps, colonies were counted, and the obtained results were presented as log CFU/g. The complete procedures and all recommendations from the standardized methodology were followed in detail. All incubations were carried out in temperature-controlled incubators (Medline Scientific, Oxfordshire, UK), and all measurements were performed in triplicate.

### 2.6. Preparation of Kefir Fortified with Encapsulated Blackberry Juice (EBJ)

Kefir was made in the Laboratory of Microbiology at the Faculty of Technology Novi Sad, the University of Novi Sad. Briefly, 6 g of commercial lyophilized kefir grains (containing *Lactococcus lactis* (ssp. *cremoris*, ssp. *lactis*, and ssp. *lactis* biovar diacetylactis), *S. thermophilus*, *Lactobacillus bulgaricus*, *L. kefir*, *L. parakefir*, and *Saccharomyces cerevisiae*) was activated in pasteurized cow milk (“Imlek”, Belgrade, Serbia) in a ratio of 1:8 (*w*/*v*) at 25 °C for 24 h to develop the inoculant. The obtained inoculants, without kefir grains, were added to the pasteurized milk and kefir fermentation was performed at 25 °C for 24 h. The obtained kefir was divided into five equal parts. The control was intact (without the addition of any compounds), while the other four parts were fortified with different concentrations of the prepared encapsulate: 1, 2.5, 5, and 7.5% (*w*/*w*). The fortification was performed on the same day that the kefir was produced in sterile conditions at the Laboratory of Microbiology. In order to avoid contamination, the process of fortification was performed using sterile bottles, which were quickly and carefully filled and immediately closed. The homogenization was performed with the shaker for 1 min, and the obtained final kefir drinks were refrigerated at 4 °C until further experimental steps.

### 2.7. Monitoring of Kefir Quality Parameters during Storage

During the storage period of 28 days at 4 °C, the control and fortified samples were monitored at the initial point (0 days) and every seven days (the total number of sampling points was 5). In order to determine the quality of the obtained kefirs, the following analyses were monitored during the storage period: color, rheological, titrable acidity, pH value, total polyphenol content, total anthocyanin content, antioxidant activity, and microbiological profiling analyses.

#### 2.7.1. Color Analysis and Influence of Encapsulated Blackberry Juice (EBJ) Fortification on Viscosity, pH, and Total Solids

Color analysis was conducted via the Minolta reflectance colorimeter described above. Color quantifications were measured at five points for each sample with a minimum of 10 readings, and the results are presented as average values using the CIE L*a*b* system.

Using a rotational viscometer (DV1 Digital Viscometer, Brookfield, WI, USA), the viscosity was determined (at 25 °C, cylindrical spindle, and a shear rate of 51 s^−1^). The equation of the power-law model (δ = K(γ)^n^) [33] for the calculation of the viscosity was used, where δ and γ demonstrate the shear stress (Pa) and the shear rate (s^−1^), respectively.

The titrable acidity and pH values of the control and fortified samples were monitored using the AOAC method (2000) and pH meter HI99161 (Hanna Instruments, Vunsakat, USA). The titrable acidity is expressed as g/100 mL in anhydrous lactic acid.

The extraction for the TPC and TAC estimation in the kefir samples was carried out according to Kabakci et al. [34]. Kefir samples (10 g) were exhaustively extracted using acidified 30 mL methanol (0.1% HCl, *v*/*v*), followed by centrifugation at 4000 rpm (Centrifuge, model EBA 21, from Hettich Zentrifugen, Tuttlingen, Germany) and the collection of supernatants. Methanol in the supernatants was evaporated at 40 °C in a rotary evaporator (Büchi Labortechnik, Flawil, Switzerland). Acidified ultrapure water (0.01% HCl, *v*/*v*) was used for dissolving the residue. The resulting extracts were used for the TPC, TAC, and antioxidant activity determination using the above-described protocols.

#### 2.7.2. Microbiological Profiling of Encapsulated Blackberry Juice (EBJ)-Fortified Kefir

In order to monitor the kefir culture profile, as well as the selected hygienic and microbiological safety parameters, a set of microbiological analyses were conducted during the complete storage period. For this purpose, 25 g of sample was suspended in 225 mL of double-concentrated Buffered Peptone Water solution (HiMedia, Mumbai, India) and mixed in a homogenizator (AES Chemunex, Barcelona, Spain). The series of dilutions were prepared, and aliquots of selected dilutions were transferred into nutrient media according to the standardized procedures. Briefly, the following ISO standardized analyses were performed: lactic acid bacteria (ISO 7889:2003) [35]; aerobic mesophilic bacteria (ISO 4833-1:2013) [29]; yeast and mold (ISO 21527-1:2008) [30]; *Escherichia coli* (ISO 7251:2005) [36]; *Listeria monocytogenes* (ISO 11290-2:2017) [37]; *Staphylococcus aureus* (ISO 6888-1:1999/AMD2:2018) [38]; *Salmonella* spp. (ISO 6579-1:2017) [39]; Enterobacteriaceae (ISO 21528-2:2017) [31]; and *Clostridium perfringens* (ISO 7937:2004) [32]. The complete procedures and all recommendations from the standardized methodology were followed in detail. All incubations were carried out in temperature-controlled incubators (Medline Scientific, Oxfordshire, UK), and all measurements were performed in triplicate.

### 2.8. Sensory Evaluation

Sensory characteristics of kefir samples were evaluated by a panel of 24 trained assessors (12 female and 12 male), with a mean age of 25 years. Kefir samples were presented in a random order in plastic cups. A nine-point hedonic scale was applied to determine the sensory acceptance of the kefir samples [40]; values from 1 to 9 represented from “dislike extremely” to “like extremely”. The evaluated attributes were taste, appearance, aroma, color, texture, and overall acceptance.

### 2.9. Statistical Analyses

All analyses were performed in triplicate and the results are presented as mean values ± standard deviations. One-way analysis of variance (ANOVA) with a Tukey’s HSD test was used to determine significant differences among the data at the significance level *p* < 0.05. Statistical analyses were carried out using the Origin 8.0 SRO software package and Microsoft Office Excel 2010.

## 3. Results and Discussion

### 3.1. Lentil Protein Isolate (LPI) and Encapsulated Blackberry Juice (EBJ)

#### 3.1.1. Physicochemical and Color Properties

**Figure 1:**
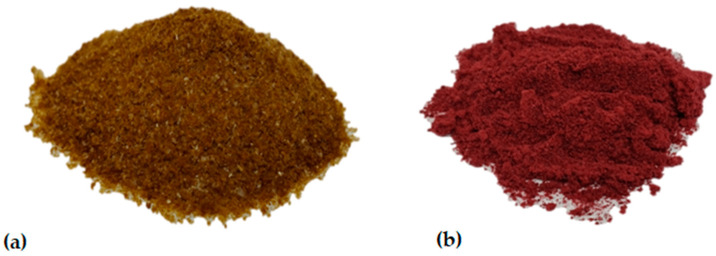
Images of (**a**) lentil protein isolate and (**b**) encapsulated blackberry juice.

A mixture of the blackberry juice and lentil protein isolate with no further ingredients was prepared and processed into juice encapsulates via freeze-drying. The general appearances of the LPI and EBJ are shown in Figure 1, and the physicochemical properties of these samples are presented in Table 1.

The water activity and moisture content are valuable quality parameters, as they determine the stability, flowability, drying efficiency, stickiness, oxidation of bioactive molecules, and potential of microbial growth on the final encapsulates. For both samples, the a_w_ values (LPI: 0.09; EBJ: 0.24) and moisture content (LPI: 1.64 g/100 g; EBJ: 2.17 g/100 g) were within the maximum a_w_ and moisture specification for dried encapsulates in the food industry, which are 0.6 and 3–4 g/100 g, respectively [41]. The obtained results suggest that the final encapsulates could be stable during storage.

Hygroscopicity is an important parameter for food encapsulation technology and presents the ability of encapsulates to absorb moisture from a relatively humid environment. Therefore, defining this parameter offers the best possibility of maintaining the best storage conditions. The hygroscopicity of encapsulates can be classified as follows: non-hygroscopic (<10%), slightly hygroscopic (10–15%), and hygroscopic (15–20%) [42]. According to this classification, LPI and EBJ could be considered non-hygroscopic and stable during storage.

Among the different physicochemical properties, the solubility and ability of proteins to bind water or oil are the most important for their usage in food applications. The obtained results of these characteristics showed that the LPI had higher solubility (56%) compared to the EBJ (29%). These results are in good agreement with the obtained results for the water-holding capacity. The WHC results showed the superior water-binding ability of the LPI compared to the EBJ. Moreover, the EBJ showed a better ability to retain oil, resulting in a higher oil-holding capacity (3.66 g/g). The higher oil-holding capacity of proteins greatly influences the shelf life and textural and other quality properties of food products [43].

Bulk density (Db) is also a significant characteristic of encapsulates. To ensure consistency during packaging, the encapsulates must meet Db criteria. Via the Db and Dt parameters, it is possible to present two parameters: the CR, which is an indicator of the flowability, and the HR, which is a measurement of the cohesiveness. According to these parameters, the obtained EBJ had fair flowability and intermediate cohesiveness. The free-flow properties of encapsulates are highly dependent on the carrier characteristics, as well as the technique applied for encapsulation [44].

Because color influences the sensory attractiveness of foods, this quality indicator is of importance for consumers, particularly for enriched products in which encapsulates are used as a colorant. The EBJ was characterized by lower lightness (L* = 16.30) and yellowness (b* = 13.90), as well as greater redness (a* = 35.80), compared to the LPI (L* = 17.90; a* = 17.75; b* = 54.05). The darker color and superior redness of the EBJ are explained by the encapsulated anthocyanins, which are the main features of blackberries. Jiménez-Aguilar et al. [45] also reported that the coordinate a* is attributed to the anthocyanin content of the fruit. In addition, color changes in food products enriched with encapsulated bioactive compounds can occur due to the formation of polymeric anthocyanins [46]. According to Weiss et al. [47], the relative amounts of flavylium cations (red or orange), quinoidal forms (blue), carbinol pseudobase (colorless), and chalcones (colorless or slightly yellowish) in equilibrium conditions might vary depending upon the pH and the anthocyanin structure.

#### 3.1.2. Morphological Properties

**Figure 2:**
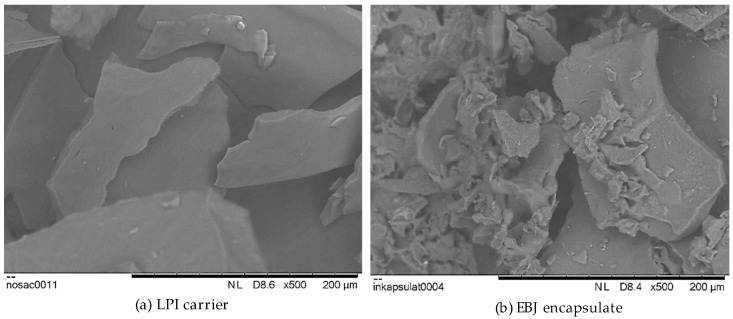
SEM imaging of surface morphology of (**a**) lentil protein isolate (LPI) and (**b**) encapsulated blackberry juice (EBJ) at a magnification of ×500.

The morphologies of the carrier (LPI) and encapsulate (EBJ) are shown in Figure 2. The carrier was similar to broken glass and had a square–rectangular structure with smooth, straight edges (Figure 2a). The reason for this structure may be in the obtaining procedure, namely in the low temperature and the lack of forces that alter the surface topology during the evaporation process [24,48]. Figure 2b shows the structure of the freeze-dried encapsulate in which the base is the described carrier with particles different in diameter, irregular shapes, pores, and wrinkles. According to Fernandes et al. [49], this structure originates from encapsulated phytochemicals and indicates the encapsulating agent’s suitability.

#### 3.1.3. Phytochemical Content and Antioxidant Properties

The encapsulation technique must ensure a high retention rate of the bioactive ingredients to effectively reach the site of action in high quantities. The encapsulation efficiency (EE) is defined as the amount of the bioactive ingredient that is effectively encapsulated in the carrier material. The EE is mainly influenced by the properties of the carrier and core materials, as well as the drying parameters. Table 2 summarizes the results of the phytochemical content and antioxidant properties of the LPI and EBJ.

According to the EE results (EE TPC = 89.68%; EE TAC = 91.12%), the combination of the freeze-drying technique and LPI as a carrier for the encapsulation of polyphenols/anthocyanins from blackberry juice was very successful. Robert et al. (2010) obtained lower values for the encapsulation of polyphenols/anthocyanins from pomegranate juice using soy proteins (76.2% and 58.5%, respectively). Dumitrascu et al. [50] also reported a lower EE of anthocyanins from cornelian cherry juice (68%) when using soy protein concentrate as the coating material and freeze-drying. Further, Diaz et al. [51] investigated the effect of wall materials on the physicochemical and antioxidant properties of blackberry juice; the authors reported that guma arabica had the highest percentage of encapsulation (71.62%), followed by maltodextrin (66.45%) and whey protein concentrate (23.33%). In this study, high encapsulating efficiencies of anthocyanins by individual coating agents and the freeze-drying technique were obtained, which is not in agreement with the assertion reported by Tao et al. [52], who reported that mixtures of coating agents in different combinations are more efficient than the individual compounds for the encapsulation of anthocyanins.

Bioactive compounds present in blackberry juice are considered to exert health-beneficial properties mainly through antioxidant activities. Currently, there is no single antioxidant assay that can accurately evaluate the contribution of bioactive compounds to the total antioxidant activity of plants or food. The amount and type of antioxidants present in biological samples, as well as the free radicals or the oxidants used in the tests, play a significant role in measuring the antioxidant activity. To evaluate the antioxidant activity in fruit juices, it is important to use different antioxidant assays, instead of relying on a single assay. In addition, results that are not seen when testing individual ingredients may vary as a result of synergistic effects and the concentration [53]. The encapsulated juice presented good antioxidant activity with a DPPH value of 1024.09 µmol TE/100 g, an RP of 547.12 µmol TE/100 g, and an ABTS of 1452.71 µmol TE/100 g.

#### 3.1.4. Microbiological Profiles

To proceed to the next steps of the experimental part, it was necessary to determine the microbiological statuses of the carrier and encapsulate used in this study. According to the values of all the selected microbiological parameters presented in Table 3, it can be concluded that there was no contamination observed in the EBJ. However, relatively low numbers of aerobic mesophilic bacteria (1.2 log CFU/g) and yeast and mold (1.4 log CFU/g) were observed in the case of the tested carrier, which was expected considering its origin (lens protein). Taking all into account, it can be concluded that the tested LPI and EBJ are acceptable for further use in the creation of fortified kefir from the microbiological point of view.

### 3.2. Kefir Fortified with Encapsulated Blackberry Juice (EBJ)

**Figure 3:**
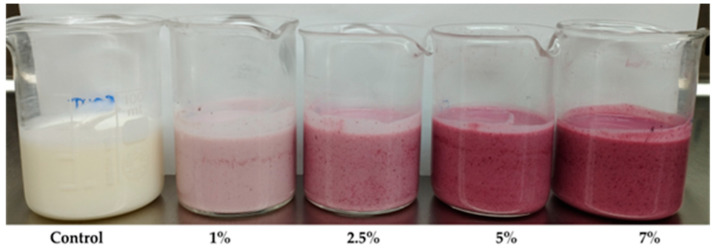
Appearances of the kefir control sample and samples with EBJ fortification at different concentration levels: 1, 2.5, 5, and 7% (*w*/*w*).

The appearances of the kefirs after fortification with EBJ at concentration levels of 1, 2.5, 5, and 7.5% (*w*/*w*) are shown in Figure 3. With the increasing encapsulate concentrations, the produced color became more intense. The introduced encapsulate was not dispersed homogeneously within the product; the appearance of stains or pigmented grains can be observed. Because the solubility of EBJ is low (29.30%, Table 1), this appearance of the kefir samples was expected.

#### 3.2.1. Influence of Encapsulated Blackberry Juice (EBJ) Kefir Fortification on Color, Titratable Acidity, and pH during Storage

Table 4 summarizes the color parameters L*, a*, b*, and C*. The L* parameter reveals that the EBJ addition significantly decreased the kefir luminosity with progressively lower values with the increasing encapsulate concentration. This observation is in agreement with the sample appearance shown in Figure 3, as well as with the anthocyanin content (as the anthocyanins gave color to the samples). The L* varied for all concentration levels during the storage period; even though these variations were statistically significant, the behavior was minimal for all samples; thus, from a technological standpoint, it should not have an impact on the visual perception.

Parameter a* was very sensitive to the EBJ addition; even at the lowest concentration (1%, *w*/*w*), the value changed from negative to positive, indicating the predominance of the red color. With the EBJ addition, the b* color parameter changed from positive to negative, indicating a predominantly blue color. Although blackberry juice has a dark-red color, at acidic pH values (pH~4.0), anthocyanins present a light-purple color. The parameter C* values, or Chroma, also followed the increase in the EBJ concentrations; the kefirs with greater anthocyanin concentrations presented higher C* values. Du and Myracle [54] also reported positive a* and negative b* values for aronia kefir; these values indicated that the product presented a bluish-red color. The authors expected the bluish-red color in the products due to the acidic environment of kefir.

Table 5 provides data on various parameters, namely the viscosity, titrable acidity, and pH values, for the kefir control sample and EBJ-fortified kefir samples over a 28-day storage period. These parameters impact the sensory attributes, safety, and product consistency. Monitoring and controlling these parameters throughout the storage process are essential for delivering a high-quality kefir product that meets consumer expectations and safety standards. The viscosity is one of the most important parameters in kefir quality assessment. It measures the thickness and flow properties of this dairy product, which are influenced by the interaction of proteins, microbes, and other components in the matrix. Viscosity impacts the mouthfeel and texture of kefir, making it a critical attribute for consumer acceptance. For quality control, monitoring the viscosity is essential to ensure consistency and meet consumer expectations. In the control sample, the viscosity increased slightly from 1.89 at day 0 to 1.93 at day 28. The 1% EBJ (*w*/*w*) sample showed a more significant increase, with the viscosity ranging from 1.91 to 2.17 Pa·s. The 2.5% EBJ (*w*/*w*) sample exhibited a similar trend, with the viscosity increasing from 1.94 to 2.14 Pa·s. Moreover, the 5% EBJ (*w*/*w*) sample displayed a consistent increase in viscosity, ranging from 1.95 to 2.23 Pa·s, while the 7.5% EBJ (*w*/*w*) sample showed a steady increase from 2.03 to 2.26 Pa·s. The typical viscosity values for kefir can range from 1.5 to 2.5 Pa·s, although these values can vary depending on factors like the fat content, fermentation time, and specific microbial strains used.

As for other fermented dairy products, acidity plays a critical role in kefir quality control. It is primarily measured as the titrable acidity, which quantifies the concentration of acids in the kefir. The specific tangy flavor and preservative properties of kefir are closely associated with its acidity. In quality control, monitoring and maintaining the titrable acidity within a specified range are crucial. Typical values for the titrable acidity in kefir usually fall within the range of 60–120 g/100 g of lactic acid, although this can vary based on regional and consumer preferences. In the case of the control samples, this value decreased from 71 to 61 g/100 g of lactic acid in 28 storage days, which reflects a significant decrease. The 1% EBJ (*w*/*w*) sample displayed relatively stable titrable acidity levels within the range of 64–71 g/100 g of lactic acid. The 2.5% EBJ (*w*/*w*) sample showed a slight decrease, ranging from 74 to 72 g/100 g of lactic acid. The 5% EBJ (*w*/*w*) sample maintained relatively stable titrable acidity levels within the range of 81–83 g/100 g of lactic acid. The 7.5% EBJ (*w*/*w*) sample exhibited a decrease in titrable acidity from 83 to 67 g/100 g of lactic acid, with a significant decrease observed on day 28.

The last but not least important parameter for kefir quality monitoring is the pH value. The pH value is a key indicator of the microbial activity in the kefir and is inversely related to the acidity, meaning that as the pH decreases, the acidity increases. Monitoring the pH value during storage helps in assessing the kefir’s overall stability and the progression of the fermentation. Typical pH values for kefir fall within the range of 4.0–4.6. In the control sample, the pH value increased from 4.21 at day 0 to 4.67 at day 28, indicating a progressive pH increase over time. The 1% EBJ (*w*/*w*) sample displayed an increase in pH from 4.12 to 4.21, with some fluctuations. The 2.5% EBJ (*w*/*w*) sample showed a gradual increase in pH, ranging from 4.01 to 4.19. The 5% EBJ (*w*/*w*) sample maintained a relatively stable pH within the range of 3.79–3.88. In contrast, the 7.5% EBJ (*w*/*w*) sample exhibited a decrease in pH from 3.81 to 4.34, with significant fluctuations and a sharp increase observed on day 28. It must be noted that monitoring the pH is important not only for the flavor but also for safety, as extreme pH values can indicate spoilage or the growth of undesirable microorganisms.

#### 3.2.2. Influence of Encapsulated Blackberry Juice (EBJ) Kefir Fortification on Phytochemical Content and Antioxidant Activity during Storage

Table 6 shows the phytochemical content and antioxidant properties of the kefir samples during storage. Increases in the TPC, TAC, and antioxidant activity are the main aims of kefir fortification. Initially, the TPC content varied from 98.64 to 181.12 mg GAE/100 g kefir, while the TAC varied from 18.25 to 44.56 mg CyGE/100 g kefir, according to the increase in the EBJ concentration added. The addition of EBJ, for all concentrations, showed a similar behavior during storage. A slight decrease in the phytochemical content with time was observed. A temporary decrease in the TPC in the kefir samples could be explained by the decomposition of polymeric phenolics in the presence of lactic acid bacteria during refrigerated storage [55]. The antioxidant activity followed the same trend. Increasing the fortification level of the EBJ led to an increase in the antioxidant activity of the kefir against DPPH and ABTS, as well as reducing power. The high increase in the antioxidant activity of the fortified kefirs was due to the presence of phenolic compounds with prominent antioxidant activity, such as anthocyanins (probably cyanidin-3-O-glucoside and cyanidin-3-O-dioxalylglucoside) and certain phenolic acids (probably ellagic acid, chlorogenic acid, caffeic acid, and gallic acid) [56]. Barretto et al. [57] investigated the stability of encapsulated and non-encapsulated anthocyanins in yogurt produced with natural dye obtained from Solanum melongena L. bark. The authors reported the greater stability of the anthocyanins during storage, as well as antioxidant activity, when added in encapsulated form in yogurt. Kabacki et al. [34] also reported a gradual degradation of anthocyanins during storage; the authors showed a negative correlation between glucose and anthocyanins during the storage of kefir fortified with anthocyanin-rich juices. Thus, among the previously mentioned factors, such as heat, light, oxygen, and pH, the differences in the anthocyanin stability may also result from variations in the sugar composition in the fermented matrices. Esatbeyoglu et al. [58] recently reported that the total anthocyanin content of water kefir made of aronia pomace and juice decreased with the fermentation (72 h). The authors reported that the kefir with aronia pomace had higher TACs (17.8 and 13.3 mg/100 mL kefir for 0 h and 72 h, respectively) compared to those with aronia juice (7.30 and 5.34 mg/100 mL kefir at 0 h and 72 h, respectively). Overall, the addition of encapsulated anthocyanins in the step after fermentation is a promising strategy for maintaining the good stability of these compounds throughout storage. Based on the 240 mL serving size (1 cup), all the produced kefirs in this study could provide more anthocyanins than the daily recommended intake, which is 12.5 mg/day/person [59].

#### 3.2.3. Influence of Encapsulated Blackberry Juice (EBJ) Kefir Fortification on Microbiological Profile during Storage

The tracking of the microbiological statuses of the kefirs over the storage time was performed on the day of kefir fortification and then every seven days until the 28th day. According to the obtained results presented in Table 7, no *L. monocytogenes*, *Salmonella* spp., *S. aureus*, *E. coli*, Enterobacteriaceae, or *Cl. pefrigens* were found in any of the kefir samples at any time of the analysis. This result is an excellent indicator of the good laboratory practice and hygiene implemented during the experiments. Also, in all samples at the starting point, the numbers of LAB 1, LAB 2, and AMB were between 7 and 8 log CFU/mL, while the numbers of yeast and mold were approximately 5 log CFU/mL. The Fermented Milks Codex [60] lists 10^4^ CFU/g as the minimum yeast content in kefir and 10^7^ CFU/g as the minimum load of total microorganisms constituting the starter culture. Thus, the obtained initial kefirs met the requirements appointed by the Fermented Milks Codex. Slightly higher numbers of AMB and yeast and mold could be noticed in all the fortified kefirs in comparison to the control kefir sample. This result is a consequence of the addition of an encapsulate and suitable carrier, which was demonstrated to carry low numbers of AMB and yeast and mold (Table 7), to the kefir during the process of fortification, resulting in increases in the total numbers of these microorganisms at the starting point. A similar situation was earlier presented by Goncu et al. [61], whose work has demonstrated that the addition of dietary fibers in kefir leads to the stimulated growth of LAB. During the storage period, slightly decreasing numbers of LAB 1, LAB2, and AMB could be noticed in all the samples. After the 28th day, the initial numbers of the mentioned groups of microorganisms were lower than the initial numbers by approximately 1–2 log CFU/mL. Contrarily, the numbers of yeast and mold increased by approximately 1 log CFU/mL in all the tested samples over time. The most intensive changes in the number of tested groups of microorganisms were observed for the samples with the addition of 5% and 7.5% of EBJ, while the samples with less EBJ added did not significantly vary from the control kefir sample. The potential reasons for the observed behavior could be not only the type and concentration of bioactive substances and the initial concentrations of individual groups of bacteria and yeast and their mutual relationship, but also an antimicrobial effect of the microbial products, as well as stimulated storage conditions for only one group of tested microorganisms. Depending on the specific bioactive substances and their concentration, fortification can lead to changes in the microbial composition [62]. Briefly, the fortification of kefir with some types of additional nutrients can either support the growth of beneficial microorganisms or potentially compete with yeast and bacteria for resources [63]. Moreover, yeast and lactic acid bacteria are sensitive to pH changes, so changes in the pH value can influence their growth and survival [12]. On the one hand, bioactive substances can have an inhibitory effect on certain microorganisms, and their encapsulated forms can enable a prolonged influence of microbial populations in kefirs. On the other hand, some bioactive compounds may act as prebiotics, providing a source of nutrients for microorganisms and encouraging the growth of specific strains [64]. Additionally, the antioxidant potential can be correlated with changes in the kefir microbial community due to a strong influence on the oxidative stress and, consequently, on the microbial viability during the storage period [64]. Considering that the obtained results indicate that higher EBJ concentrations have a stronger influence on the microbial community, specific concentrations of these compounds can be a crucial factor and the first step in further determining the influences on kefir’s microbial composition during storage.

The exact influence of kefir fortification with bioactive substances on the yeast and bacteria concentrations will vary depending on the specific ingredients used and the microbial community in the kefir. To better understand these effects, it is essential to conduct controlled experiments with different concentrations of bioactive substances and monitor changes in the microbial population over time.

#### 3.2.4. Influence of Encapsulated Blackberry Juice (EBJ) Kefir Fortification on Sensory Attributes

**Figure 4:**
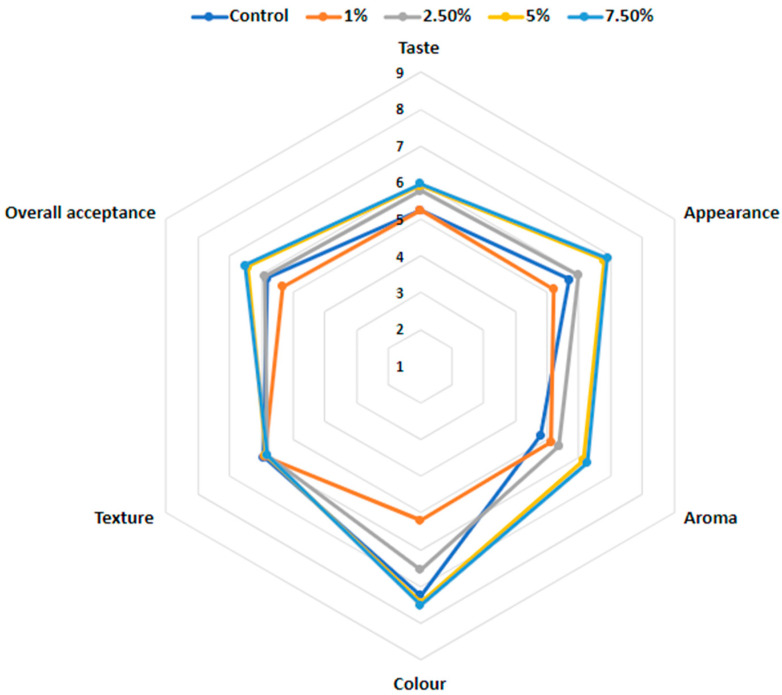
Sensory evaluation of control kefir and kefir fortified with encapsulated blackberry juice (EBJ) in concentrations of 1, 2.5, 5, and 7.5% (*w*/*w*).

The sensory evaluation showed that different percentages of EBJ strongly modified the sensory attributes of the kefir (Figure 4). The panelists demonstrated a clear preference for the kefir fortified with EBJ at greater concentrations. The most preferred samples by the panelists were the kefir containing 7.5% EBJ (6.50 overall acceptability score) GAP, followed by the kefir containing 5% EBJ (6.42 overall acceptability score). The least preferred concentration among all the kefirs was 1% (5.34 score for overall acceptability). A similar trend was reported by Šeregelj et al. [65] when evaluating the sensory acceptance of yogurt fortified with red pepper waste encapsulate. In addition, similar results of acceptance were reported by Rubio et al. [66] for colored yogurts using colorants extracted from grape pomace and jabuticaba by-products and encapsulated via spray-drying using brewer’s spent yeast as the carrier. In the study by Domingos Pinto et al. [67], the panelists showed good acceptance for all the tested attributes of yogurt colored with anthocyanin-rich pigment prepared from jabuticaba skin. The authors noted that natural pigments have the potential to be a substitute synthetic color additive to produce mixed-berry yogurts.

## 4. Conclusions

This study specifies that bioactive compounds (polyphenols and anthocyanins) from blackberry juice were successfully encapsulated with LPI using a freeze-drying technique. Kefir fortified with EBJ in different concentrations displayed enhanced antioxidant properties, making it a promising functional food ingredient. The microbiological analysis confirmed the safety of the carrier and encapsulate, indicating their suitability for use in kefir fortification. Moreover, the stability of the fortified products was confirmed by monitoring the viscosity, titrable acidity, and pH values. The sensory evaluation revealed that higher EBJ concentrations in kefir were preferred by the panelists, demonstrating the potential for product diversification.

As the main milestones of this study, the high encapsulation efficiency, safety assurance, and enhanced antioxidant properties and sensory attributes of the final products can be emphasized. The study’s sensory evaluation showed that the addition of EBJ at higher concentrations led to increased overall acceptability, indicating the potential for creating appealing kefir variants. Furthermore, the encapsulated kefir with improved antioxidant activity offers the potential for the development of functional food products with health benefits. Future research should focus on enhancing the stability of encapsulated bioactive compounds during storage, potentially through the use of modified encapsulation techniques. Further studies can explore consumer preferences and demands for encapsulated kefir products, leading to tailored offerings for different market segments. Moreover, investigating the specific health benefits associated with the enhanced antioxidant properties of encapsulated kefir could open doors to functional food products that target particular health concerns. Future research should also address the environmental aspects of encapsulation materials (i.e., the potential use of encapsulation materials isolated from different plant by-products). In summary, this study presents a promising approach to enhancing the quality and functionality of kefir through encapsulation. While challenges related to the stability and appearance should be addressed, the potential for creating appealing and health-beneficial kefir products is evident, offering opportunities for future research and product development.

## Figures and Tables

**Table 1:** Physicochemical and color properties of lentil protein isolate (LPI) and encapsulated blackberry juice (EBJ).

Properties	Lentil Protein Isolate (LPI)	Encapsulated Blackberry Juice (EBJ)
Water activity (a_w_)	0.09 ± 0.00	0.24 ± 0.00
Moisture content (g/100 g)	1.64 ± 0.00	2.17 ± 0.01
Hygroscopic moisture (g/100 g)	2.71 ± 0.01	2.98 ± 0.02
Solubility (%)	56.50 ± 0.26	29.30 ± 0.26
Water-holding capacity (WHC) (g/g)	1.72 ± 0.06	1.55 ± 0.03
Oil-holding capacity (OHC) (g/g)	1.48 ± 0.02	3.66 ± 0.36
Bulk density (Db) (g/mL)	0.91 ± 0.00	0.87 ± 0.00
Tapped density (Dt) (g/mL)	1.79 ± 0.00	1.10 ± 0.00
Compressibility ratio (CR)	49.16 ± 0.00	20.91 ± 0.00
Hausner ratio (HR)	1.97 ± 0.00	1.26
Flowability	Very bad	Fair
Cohesiveness	High	Intermediate
L*	17.90 ± 0.02	16.30 ± 0.04
a*	17.75 ± 0.02	35.80 ± 0.04
b*	54.05 ± 0.03	13.90 ± 0.03
C*	56.89 ± 0.02	38.40 ± 0.04

**Table 2:** Functional properties of lentil protein isolate (LPI) and encapsulated blackberry juice (EBJ). nd—not detected.

Properties	Lentil Protein Isolate (LPI)	Encapsulated Blackberry Juice (EBJ)
Total polyphenol content (TPC) (mg GAE/100 g)	124.08 ± 7.76	411.06 ± 9.87
Total anthocyanin content (TAC) (mg CyGE/100 g)	nd	323.81 ± 14.65
EE TPC (%)	-	89.68 ± 2.14
EE TAC (%)	-	91.12 ± 4.55
DPPH (µmol TE/100 g)	116.41 ± 3.68	1024.09 ± 14.11
RP (µmol TE/100 g)	75.99 ± 4.56	547.12 ± 17.22
ABTS (µmol TE/100 g)	214.03 ± 13.57	1452.71 ± 28.30

**Table 3:** Microbiological profiles of lentil protein isolate (LPI) and encapsulated blackberry juice (EBJ).

Microbiological Parameter Log CFU/g	Lentil Protein Isolate (LPI)	Encapsulated Blackberry Juice (EBJ)
Aerobic mesophilic bacteria	1.20 ± 0.54	<1
Yeast and mold	1.40 ± 0.00	<1
Enterobacteriaceae	<1	<1
*Clostridium perfringens*	<1	<1

**Table 4:** Color properties during storage.

Kefir Sample	Parameter	Storage Period (Days)				
		0	7	14	21	28
Control	L*	97.20 ± 1.54 ^a^	98.20 ± 1.11 ^a^	95.14 ± 1.20 ^a^	96.20 ± 0.24 ^a^	97.10 ± 1.65 ^b^
	a*	−1.60 ± 0.20 ^a^	−1.91 ± 0.01 ^b^	−2.00 ± 0.01 ^b^	−2.31 ± 0.00 ^c^	−2.40 ± 0.01 ^c^
	b*	3.31 ± 0.04 ^a^	3.45 ± 0.01 ^a^	3.41 ± 0.0 ^a^	3.36 ± 0.00 ^a^	3.29 ± 0.04 ^a^
	Cr*	3.68 ± 0.25 ^a^	3.94 ± 0.02 ^b^	3.95 ± 0.01 ^b^	4.08 ± 0.00 ^b^	4.07 ± 0.05 ^b^
1%	L*	74.80 ± 1.31 ^a^	74.51 ± 1.49 ^a^	76.50 ± 1.27 ^a^	75.11 ± 1.14 ^a^	74.20 ± 1.61 ^a^
	a*	10.72 ± 0.33 ^b^	9.85 ± 0.24 ^a^	11.31 ± 0.21 ^c^	10.62 ± 0.17 ^b^	11.11 ± 0.08 ^c^
	b*	−4.51 ± 0.02 ^a^	−4.42 ± 0.00 ^a^	−4.91 ± 0.17 ^b^	−4.42 ± 0.02 ^a^	−4.64 ± 0.13 ^a^
	Cr*	11.61 ± 0.55 ^b^	10.80 ± 0.25 ^a^	12.33 ± 0.40 ^c^	11.50 ± 0.20 ^b^	12.04 ± 0.34 ^c^
2.5%	L*	62.53 ± 1.32 ^b^	59.11 ± 1.07 ^a^	62.50 ± 0.99 ^b^	62.52 ± 1.00 ^b^	60.31 ± 0.56 ^a,b^
	a*	21.90 ± 1.60 ^a^	22.32 ± 2.04 ^a^	22.31 ± 0.74 ^a^	22.40 ± 1.08 ^a^	22.32 ± 0.27 ^a^
	b*	−11.20 ± 0.63 ^a,b^	−12.11 ± 1.65 ^c^	−12.10 ± 0.00 ^c^	−10.79 ± 0.19 ^a^	−11.62 ± 0.68 ^b,c^
	Cr*	24.60 ± 2.25 ^a^	25.39 ± 3.70 ^a^	25.38 ± 0.75 ^a^	24.86 ± 1.30 ^a^	25.16 ± 1.00 ^a^
5%	L*	43.72 ± 1.84 ^b^	40.50 ± 0.53 ^a^	43.75 ± 0.00 ^b^	41.71 ± 1.51 ^a,b^	41.00 ± 0.02 ^a^
	a*	33.86 ± 0.64 ^a^	34.11 ± 0.35 ^a^	33.89 ± 0.65 ^a^	33.25 ± 0.21 ^a^	33.41 ± 0.18 ^a^
	b*	−11.52 ± 0.00 ^a^	−11.51 ± 0.07 ^a^	−11.42 ± 0.18 ^a^	−11.44 ± 0.06 ^a^	−10.99 ± 0.02 ^a^
	Cr*	35.77 ± 0.65 ^a^	34.55 ± 0.42 ^a^	35.77 ± 0.85 ^a^	35.16 ± 0.27 ^a^	35.17 ± 0.20 ^a^
7.5%	L*	34.30 ± 1.84 ^a^	36.21 ± 0.00 ^b^	37.92 ± 0.91 ^b^	33.45 ± 0.21 ^a^	33.72 ± 1.17 ^a^
	a*	45.00 ± 0.01 ^b,c^	43.54 ± 1.11 ^a,b^	41.78 ± 0.97 ^a^	47.31 ± 1.61 ^c^	44.12 ± 0.40 ^b^
	b*	−10.42 ± 0.65 ^a^	−11.11 ± 0.07 ^a^	−10.82 ± 1.96 ^a^	−10.36 ± 0.75 ^a^	−10.47 ± 0.07 ^a^
	Cr*	46.19 ± 0.66 ^b,c^	44.94 ± 1.19 ^a^	43.16 ± 2.02 ^a^	48.43 ± 2.40 ^b,c^	45.35 ± 0.05 ^a,b^

L* represents lightness (L* = 0 (black) and L* = 100 (white)), a* represents red and green color coordinates (−a* = greenness and +a* = redness), while b* represents yellow and blue color coordinates (−b* = blueness and +b* = yellowness); Cr^*^—Chroma. Different letters in the same row indicate significant differences among samples at *p* ≤ 0.05.

**Table 5:** Viscosity (Pa·s), titrable acidity (g/100 g of lactic acid), and pH of kefirs during storage.

Kefir Sample	Parameters	Storage Period (Days)				
		0	7	14	21	28
Control	Viscosity	1.89 ± 0.01 ^a^	1.91 ± 0.04 ^a^	1.87 ± 0.00 ^a^	1.91 ± 0.52 ^a^	1.93 ± 0.11 ^a^
	Titrable acidity	71.00 ± 1.5 ^c^	69.00 ± 0.56 ^c^	69.00 ± 0.00 ^c^	64.50 ± 0.56 ^b^	61.00 ± 0.00 ^a^
	pH	4.21 ± 0.02 ^a^	4.27 ± 0.00 ^a^	4.31 ± 0.56 ^a,b^	4.47 ± 0.07 ^b,c^	4.67 ± 0.05 ^c^
1%	Viscosity	1.91 ± 0.06 ^a^	2.04 ± 0.04 ^b^	2.03 ± 0.10 ^b^	2.07 ± 0.16 ^b,c^	2.17 ± 0.56 ^c^
	Titrable acidity	71.00 ± 1.00 ^b^	70.00 ± 0.00 ^b^	69.00 ± 0.00 ^b^	69.00 ± 1.0 ^b^	64.00 ± 0.56 ^a^
	pH	4.12 ± 0.05 ^a^	4.17 ± 0.00 ^a^	4.23 ± 0.56 ^a^	4.27 ± 0.14 ^a^	4.21 ± 0.04 ^a^
2.5%	Viscosity	1.94 ± 0.00 ^a^	2.10 ± 0.24 ^b^	2.12 ± 0.17 ^b^	2.12 ± 0.56 ^b^	2.14 ± 0.41 ^b^
	Titrable acidity	74.00 ± 2.00 ^a^	74.00 ± 1.00 ^a^	73.00 ± 0.00 ^a^	72.00 ± 1.00 ^a^	72.00 ± 0.00 ^a^
	pH	4.01 ± 0.03 ^a^	4.08 ± 0.07 ^a^	4.17 ± 0.01 ^a^	4.20 ± 0.00 ^a^	4.19 ± 0.34 ^a^
5%	Viscosity	1.95 ± 0.02 ^a^	2.15 ± 0.01 ^b^	2.21 ± 0.17 ^b^	2.23 ± 0.23 ^b^	2.23 ± 0.00 ^b^
	Titrable acidity	81.00 ± 1.00 ^a^	82.00 ± 0.00 ^a^	82.00 ± 0.00 ^a^	83.00 ± 0.00 ^a^	82.00 ± 0.00 ^a^
	pH	3.88 ± 0.10 ^a^	3.79 ± 0.09 ^a^	3.81 ± 0.03 ^a^	3.84 ± 0.51 ^a^	3.82 ± 0.07 ^a^
7.5%	Viscosity	2.03 ± 0.19 ^a^	2.21 ± 0.11 ^b^	2.19 ± 0.25 ^b^	2.25 ± 0.41 ^b^	2.26 ± 0.09 ^b^
	Titrable acidity	83.00 ± 0.50 ^b,c^	84.00 ± 2.00 ^c^	81.00 ± 0.00 ^b,c^	79.00 ± 0.00 ^b^	67.00 ± 1.00 ^a^
	pH	3.81 ± 0.00 ^a^	3.64 ± 0.52 ^a^	3.74 ± 0.14 ^a^	4.18 ± 0.21 ^b^	4.34 ± 0.05 ^b^

Different letters in the same row indicate significant differences among samples at *p* ≤ 0.05.

**Table 6:** Functional properties of kefir fortified with encapsulated blackberry juice (EBJ).

Kefir Sample	Parameter	Storage Period (Days)				
		0	7	14	21	28
Control	TPC	nd	nd	nd	nd	nd
	TAC	nd	nd	nd	nd	nd
	DPPH	20.01 ± 0.06 ^b^	20.65 ± 1.02 ^b^	18.14 ± 0.26 ^a^	18.11 ± 1.35 ^a^	17.41 ± 0.23 ^a^
	RP	nd	nd	nd	nd	nd
	ABTS	44.11 ± 2.15 ^b^	45.18 ± 2.13 ^b^	41.56 ± 1.101 ^a^	40.35 ± 0.89 ^a^	40.15 ± 1.65 ^a^
1%	TPC	98.64 ± 0.78 ^a^	99.11 ± 2.14 ^a^	98.96 ± 3.58 ^a^	98.05 ± 2.14 ^a^	98.71 ± 3.44 ^a^
	TAC	18.25 ± 0.63 ^a^	18.12 ± 2.26 ^a^	19.21 ± 1.14 ^b^	19.06 ± 2.17 ^a,b^	18.11 ± 1.09 ^a^
	DPPH	181.16 ± 9.81 ^a^	180.99 ± 10.12 ^a^	176.58 ± 13.69 ^a^	179.36 ± 9.11 ^a^	180.47 ± 21.07 ^a^
	RP	10.13 ± 0.51 ^a^	11.79 ± 0.65 ^a^	10.34 ± 0.74 ^a^	11.55 ± 0.98 ^a^	11.10 ± 0.36 ^a^
	ABTS	265.04 ± 13.44 ^c^	249.11 ± 7.29 ^b^	233.15 ± 10.41 ^a^	251.29 ± 17.21 ^b^	248.15 ± 16.54 ^b^
2.5%	TPC	126.42 ± 9.15 ^a,b^	129.87 ± 5.13 ^b^	125.30 ± 8.91 ^a,b^	125.52 ± 11.60 ^a,b^	123.10 ± 9.56 ^a^
	TAC	21.62 ± 1.51 ^b^	21.98 ± 3.22 ^b^	20.87 ± 2.09 ^a^	20.91 ± 2.86 ^a,b^	20.44 ± 1.13 ^a^
	DPPH	205.23 ± 14.63 ^a^	211.58 ± 10.55 ^a^	209.78 ± 13.17 ^a^	206.34 ± 9.15 ^a^	204.19 ± 5.68 ^a^
	RP	12.78 ± 0.98 ^b^	13.71 ± 1.63 ^c^	11.25 ± 0.44 ^a^	13.41 ± 0.97 ^b,c^	13.55 ± 1.11 ^c^
	ABTS	287.10 ± 22.65 ^a,b^	292.51 ± 13.74 ^b^	276.77 ± 13.81 ^a^	296.86 ± 15.19 ^b^	289.16 ± 5.00 ^a,b^
5%	TPC	163.71 ± 9.08 ^a^	160.12 ± 2.50 ^a^	161.41 ± 3.82 ^a^	160.99 ± 7.85 ^a^	159.84 ± 5.06 ^a^
	TAC	38.40 ± 1.25 ^b^	34.13 ± 2.81 ^a^	34.54 ± 3.96 ^a^	33.41 ± 2.21 ^a^	34.07 ± 4.20 ^a^
	DPPH	256.08 ± 5.01 ^a^	249.66 ± 11.40 ^a^	247.35 ± 6.39 ^a^	243.17 ± 16.58 ^a^	244.96 ± 7.89 ^a^
	RP	17.99 ± 3.47 ^d^	15.81 ± 2.07 ^b,c^	16.33 ± 2.50 ^c^	15.36 ± 1.18 ^b^	14.18 ± 2.00 ^a^
	ABTS	328.10 ± 15.06 ^a^	324.45 ± 11.43 ^a^	313.06 ± 12.98 ^a^	310.55 ± 17.62 ^a^	312.69 ± 5.14 ^a^
7.5%	TPC	181.12 ± 9.10 ^a^	179.57 ± 8.97 ^a^	175.13 ± 12.01 ^a^	174.41 ± 6.33 ^a^	172.08 ± 6.25 ^a^
	TAC	44.56 ± 2.96 ^b^	43.91 ± 0.81 ^a,b^	44.63 ± 1.17 ^b^	42.09 ± 2.74 ^a^	43.36 ± 1.25 ^a,b^
	DPPH	221.41 ± 11.52 ^b^	215.31 ± 14.52 ^a,b^	208.12 ± 17.65 ^a^	208.41 ± 13.58 ^a^	205.10 ± 6.28 ^a^
	RP	17.97 ± 1.36 ^d^	16.95 ± 2.83 ^c^	14.25 ± 1.08 ^a^	15.35 ± 2.97 ^b^	14.51 ± 1.88 ^a^
	ABTS	301.20 ± 28.15 ^b^	295.42 ± 15.14 ^b^	276.77 ± 13.81 ^a^	280.77 ± 19.09 ^a^	280.10 ± 14.53 ^a^

Different letters in the same row indicate significant differences among samples at *p* ≤ 0.05; nd—not detected.

**Table 7:** Microbiological profiling of kefirs during storage (results are represented as log CFU/mL for all quantification analyses, while results of detection of *L. monocytogenes* and *Salmonella* spp. are presented as detected/not detected).

Kefir Sample	Parameter	Storage Period (Days)				
		0	7	14	21	28
Control	LAB 1	8.02 ± 0.33 ^c^	8.10 ± 0.74 ^c^	7.80 ± 0.33 ^b^	7.40 ± 0.64 ^b^	6.90 ± 0.00 ^a^
	LAB 2	7.10 ± 0.00 ^d^	6.90 ± 0.56 ^c^	6.80 ± 0.00 ^c^	6.50 ± 0.56 ^a^	6.10 ± 0.33 ^a^
	AMB	7.20 ± 0.64 ^d^	7.00 ± 0.00 ^c^	7.00 ± 0.33 ^c^	6.70 ± 0.37 ^b^	6.50 ± 0.00 ^a^
	Yeast and mold	4.80 ± 0.37 ^a^	4.90 ± 0.33 ^a^	4.90 ± 0.56 ^a^	5.30 ± 0.00 ^b^	5.70 ± 0.74 ^c^
	*L. monocytogenes*	nd				
	*Salmonella* spp.					
	*S. aureus*	<1				
	*E. coli*					
	Enterobacteriaceae					
	*Cl. perfringens*					
1%	LAB 1	8.20 ± 0.00 ^d^	8.00 ± 0.00 ^c,d^	7.70 ± 0.74 ^c^	7.10 ± 0.33 ^b^	6.80 ± 0.27 ^a^
	LAB 2	7.10 ± 0.33 ^c^	6.80 ± 0.33 ^b^	6.60 ± 0.27 ^b^	6.20 ± 0.74 ^a^	5.90 ± 0.00 ^a^
	AMB	7.50 ± 0.56 ^c^	7.50 ± 0.74 ^c^	7.20 ± 0.00 ^b,c^	6.90 ± 0.64 ^a,b^	6.60 ± 0.33 ^a^
	Yeast and mold	5.10 ± 0.00 ^a^	5.20 ± 0.64 ^a^	5.50 ± 0.33 ^b^	5.70 ± 0.37 ^b^	6.10 ± 0.56 ^c^
	*L. monocytogenes*	nd				
	*Salmonella* spp.					
	*S. aureus*	<1				
	*E. coli*					
	Enterobacteriaceae					
	*Cl. perfringens*					
2.5%	LAB 1	8.20 ± 0.00 ^c^	8.10 ± 0.74 ^c^	7.80 ± 0.33 ^b,c^	7.40 ± 0.00 ^a,b^	7.00 ± 0.64 ^a^
	LAB 2	7.00 ± 0.00 ^c^	6.80 ± 0.33 ^b,c^	6.50 ± 1.32 ^b^	6.00 ± 0.00 ^a^	5.70 ± 0.33 ^a^
	AMB	7.70 ± 0.64 ^c^	7.60 ± 0.00 ^c^	7.10 ± 0.37 ^b^	6.80 ± 0.32 ^a,b^	6.50 ± 0.33 ^a^
	Yeast and mold	5.20 ± 0.56 ^a^	5.30 ± 0.74 ^a^	5.70 ± 0.64 ^c^	6.00 ± 0.74 ^c,d^	6.30 ± 0.00 ^d^
	*L. monocytogenes*	nd				
	*Salmonella* spp.					
	*S. aureus*	<1				
	*E. coli*					
	Enterobacteriaceae					
	*Cl. perfringens*					
5%	LAB 1	8.10 ± 0.33 ^d^	7.60 ± 0.27 ^c^	7.10 ± 0.00 ^b^	6.70 ± 0.00 ^a^	6.50 ± 0.33 ^a^
	LAB 2	7.00 ± 0.33 ^c^	6.80 ± 0.00 ^c^	6.40 ± 0.37 ^b^	6.00 ± 0.74 ^a^	5.80 ± 0.37 ^a^
	AMB	7.90 ± 0.0 ^d^	7.40 ± 0.64 ^c^	6.90 ± 0.33 ^b^	6.70 ± 0.64 ^a,b^	6.50 ± 0.74 ^a^
	Yeast and mold	5.50 ± 0.00 ^a^	5.80 ± 0.74 ^a,b^	6.10 ± 0.00 ^b^	6.10 ± 0.33 ^b^	6.50 ± 1.32 ^c^
	*L. monocytogenes*	nd				
	*Salmonella* spp.					
	*S. aureus*	<1				
	*E. coli*					
	Enterobacteriaceae					
	*Cl. perfringens*					
7.5%	LAB 1	8.10 ± 0.33 ^d^	7.40 ± 0.00 ^c^	7.10 ± 0.64 ^c^	6.60 ± 0.00 ^b^	6.20 ± 0.33 ^a^
	LAB 2	7.00 ± 0.00 ^d^	6.50 ± 0.56 ^c^	6.20 ± 0.00 ^b,c^	6.00 ± 0.74 ^a,b^	5.80 ± 1.32 ^a^
	AMB	8.10 ± 0.37 ^e^	7.60 ± 0.37 ^d^	7.10 ± 0.00 ^c^	6.50 ± 0.74 ^b^	6.10 ± 0.00 ^a^
	Yeast and mold	5.90 ± 0.00 ^a^	6.30 ± 0.74 ^b^	6.60 ± 0.33 ^b,c^	6.80 ± 0.33 ^c^	6.90 ± 0.64 ^c^
	*L. monocytogenes*	nd				
	*Salmonella* spp.					
	*S. aureus*	<1				
	*E. coli*					
	Enterobacteriaceae					
	*Cl. perfringens*					

LAB 1—*Lactobacillus* species; LAB 2—*Streptococcus* and *Lactococcus* species; AMB—aerobic mesophilic bacteria; nd—not detected. Different letters in the same row indicate significant differences among samples at *p* ≤ 0.05.

## Data Availability

Data are contained within the article.
